# How incorporation of scents could enhance immersive virtual experiences

**DOI:** 10.3389/fpsyg.2014.00736

**Published:** 2014-07-17

**Authors:** Matthieu Ischer, Naëm Baron, Christophe Mermoud, Isabelle Cayeux, Christelle Porcherot, David Sander, Sylvain Delplanque

**Affiliations:** ^1^Swiss Center for Affective Sciences, University of GenevaGeneva, Switzerland; ^2^Department of Psychology, University of GenevaGeneva, Switzerland; ^3^Department of Medicine, University of GenevaGeneva, Switzerland; ^4^Firmenich, S.A.Geneva, Switzerland

**Keywords:** virtual reality, olfactory display, olfactometer, emotion, scent

## Abstract

Under normal everyday conditions, senses all work together to create experiences that fill a typical person's life. Unfortunately for behavioral and cognitive researchers who investigate such experiences, standard laboratory tests are usually conducted in a nondescript room in front of a computer screen. They are very far from replicating the complexity of real world experiences. Recently, immersive virtual reality (IVR) environments became promising methods to immerse people into an almost real environment that involves more senses. IVR environments provide many similarities to the complexity of the real world and at the same time allow experimenters to constrain experimental parameters to obtain empirical data. This can eventually lead to better treatment options and/or new mechanistic hypotheses. The idea that increasing sensory modalities improve the realism of IVR environments has been empirically supported, but the senses used did not usually include olfaction. In this technology report, we will present an odor delivery system applied to a state-of-the-art IVR technology. The platform provides a three-dimensional, immersive, and fully interactive visualization environment called “Brain and Behavioral Laboratory—Immersive System” (BBL-IS). The solution we propose can reliably deliver various complex scents during different virtual scenarios, at a precise time and space and without contamination of the environment. The main features of this platform are: (i) the limited cross-contamination between odorant streams with a fast odor delivery (< 500 ms), (ii) the ease of use and control, and (iii) the possibility to synchronize the delivery of the odorant with pictures, videos or sounds. How this unique technology could be used to investigate typical research questions in olfaction (e.g., emotional elicitation, memory encoding or attentional capture by scents) will also be addressed.

## Introduction

The replication of everyday life environments in laboratory experiments is crucial in behavioral sciences because it directly improves the ecological validity of the results, especially when subtle and complex interactions are concerned (Spooner and Pachana, 2006). For instance, in affective sciences, many theories postulate that the elicitation and the differentiation of emotions are determined by continuous and recursive evaluations of events (see Ellsworth and Scherer, 2003 for an overview on appraisal models of emotions). Evaluation of the environment through smell, taste, sight, hearing, touch, temperature, and balance perception contributes to the extraordinary changeability and the high degree of qualitative differentiation of emotional experiences, as well as individual differences in emotional reactions. Consequently, being able to simulate a rich environment is a key point to investigate a large variety of affective behaviors. However, standard laboratory tests in humans are typically conducted in nondescript rooms in front of two-dimensional environments displayed on flat computer screens and are very far from replicating the complexity of real world experiences.

Advances in Immersive Virtual Reality (IVR) technologies have recently opened a new range of possibilities in empirical research. IVR technologies provide virtual environments that mimic the complexity of the real world and at the same time grant scientists with many control and monitoring capabilities. It has become a promising framework to immerse people into a close-to-reality environment that involves more human senses. The capacity to completely control the environment fulfills the experimental criteria required in many behavioral sciences. Owing to the latest advancement in computer technologies, the subject's immersion in a three-dimensional (3D) experimental scenario is constantly improved. Consequently, the “sense of presence”^1^ that leads to a direct engagement from the subject in interactivity with the 3D world is increasing. These close-to-reality experiences could possess a considerable potential in research, either to obtain better treatment options for people showing behavioral and cognitive deficits or to investigate fundamental hypotheses.

To date, real everyday life auditory and visual perceptions can be almost perfectly replicated in IVR environments. Since senses work together to create the overall sensory experience, increasing the quality of the represented environment as well as implementing more senses remain worth pursuing (Nakamoto et al., 2008). This idea has already been empirically supported (Dinh et al., 1999), but it almost never includes chemoperception (Craig et al., 2009). Because olfaction is more complex to implement and control, its uses in IVR environments remain more the exception than the rule.

Several attempts have been made to implement controlled scent delivery systems (olfactory display^2^, OD) in virtual reality environments (e.g., Richard et al., 2006). For instance, Tortell et al. (2007) used a system able to deliver four different odorants by evaporation of different chemical compounds presented on scented collars. With this methodology, the authors brought experimental elements showing that presentation of scents is a promising method by which a user's attention is devoted to the IVR environment exploration, heightening their sense of presence. However, to obtain a reliable and controlled olfactory stimulation, the OD should satisfy very important constraints, such as being able to produce reproducible releases of various kinds of compounds over multiple trials, without contamination from one trial to the other, at known time, localization and strength, and without additional noise or tactile stimulations in the nose.

So far, standard olfactory delivery systems do not propose a rich repertoire of compounds, which limits the variety and the subtlety of situations the participant can be exposed to. In order to avoid this issue, available systems (Yamanaka et al., 2002; Weiling et al., 2010) create blends of odors by mixing basic predefined sets of odors. Albeit ingenious, these solutions cannot propose a rich variety of realistic odors that are often composed of thousands of different molecules. Exception to this olfactory poverty exists. For example, Sezille et al. (2013) developed a portable OD dedicated to fMRI experiments, which satisfies most of the constraints described above and can deliver up to 15 odorants. Nakamoto and Minh (2007) designed an OD which can deliver up to 30 odorants at constant flow rate. In this configuration, the main tubes manifold (along with the bank of odorants) and the solenoid valves are attached together. As the noise of the solenoid valves could give clues to the participant about the delivery of odorants, this solution requires using long distance common odorant-bearing tubes or wearing additional headphones. These constraints increase the risks of contamination of one odorant by remaining traces of the preceding one (i.e., cross-contamination) or the weight and the number of apparatuses users should wear. Since unencumbering systems are important in IVR, Yanagida et al. (2004) proposed an OD that does not require the user to attach anything on the head. The main device involves an “air cannon” which projects scented air puffs near the user's nose. In order not to deflect the trajectory of the scented air puff, ventilation and air extraction are not integrated in the display. Unfortunately, this technical solution increases the likelihood of odor contamination in the ambient air. Users had to limit the use of this display with four low-concentrated scents delivered with short emissions, which could thus sometimes be undetectable to users. Furthermore, a contamination between odorant sources in the “air cannon” at continuous use compromised the reproducible releases of various kinds of compounds over multiple trials. Sato et al. (2009) showed that synchronizing the delivery of odors with the user's breathing pattern could prevent ambient contamination. However, the disadvantage of the setup is that users have to stay still and close to the fixed OD, which largely complicates its implementation in IVR environments where head movements, at least, should not be restricted. Yamada et al. (2006) developed another miniaturized system to be worn by the participant in an outdoor environment. This OD can deliver 3 different odors at different strengths according to a virtual “*odor field*,” but the variation of the odorant's strength is mainly controlled by an increase of the airflow. The main disadvantage associated with such a design is the possible changes in tactile sensations in the nose (due to airflow fluctuations) that are irrelevant to the odorant perception. Lastly, latencies between the order to deliver an odorant and its effective delivery to the nose (see also Narumi et al., 2011 for another head mounted OD) are often not strictly controlled or reported (Brkic et al., 2009; Ramic-Brkic and Chalmers, 2010) and it appears very difficult to rapidly and dynamically adjust the amount/intensity of odor according to the recipients' needs.

In this technology report, we will present an odor delivery solution applied to a state-of-the-art IVR technology that provides a 3D, immersive, and fully interactive visualization environment called BBL-IS (Brain and Behavior Laboratory—Immersive System). After exposing the basic principles of the system, we will present several studies that demonstrate its efficiency to deliver a large number of different odorants in the virtual environment: (i) in total safety for the subjects, (ii) reliably and in a reproducible manner, at a low and constant flow rate among subjects and without other perceptible changes (i.e., noise or tactile), (iii) with a limited cross-contamination between odorant streams, and (iv) with an easily and controllable interface. How this unique technology could be used to investigate typical research questions in olfaction (e.g., emotional elicitation, memory encoding or attentional capture by scents) will also be addressed.

## Materials and methods

### The olfactory display

#### Design

**Figure 1:**
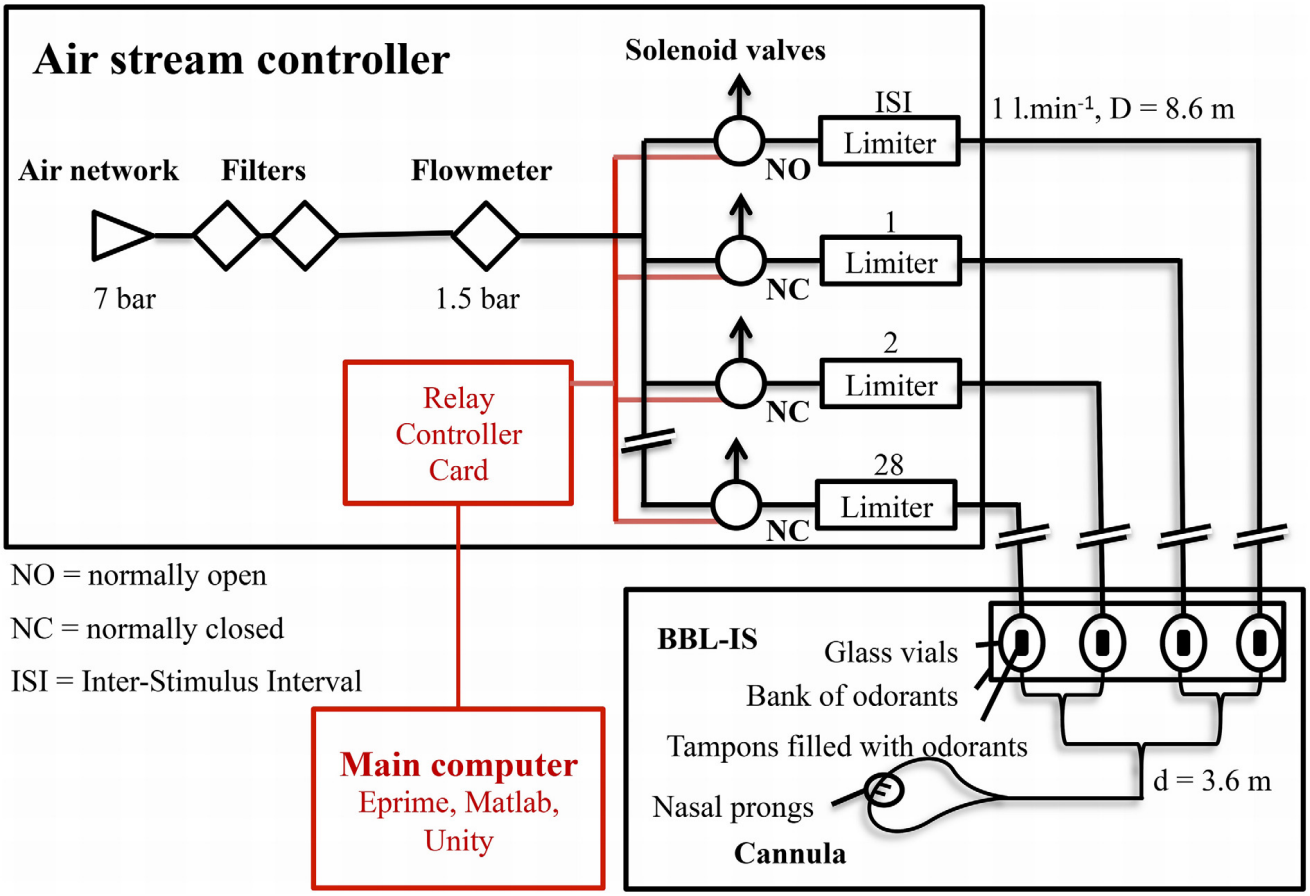
**Schematic diagram of the OD**. For clarity reasons, channels 3 to 27 are not represented.

The OD is based on a series of 32 computer-controlled solenoid valves. A schematic diagram of the OD is presented in Figure 1. Individual solenoid valves are numerated from 1 to 28, the four remaining valves being attributed to air delivery during inter-stimulus intervals (ISI), and CO_2_ at different concentrations (not described in this report).

**Figure 2:**
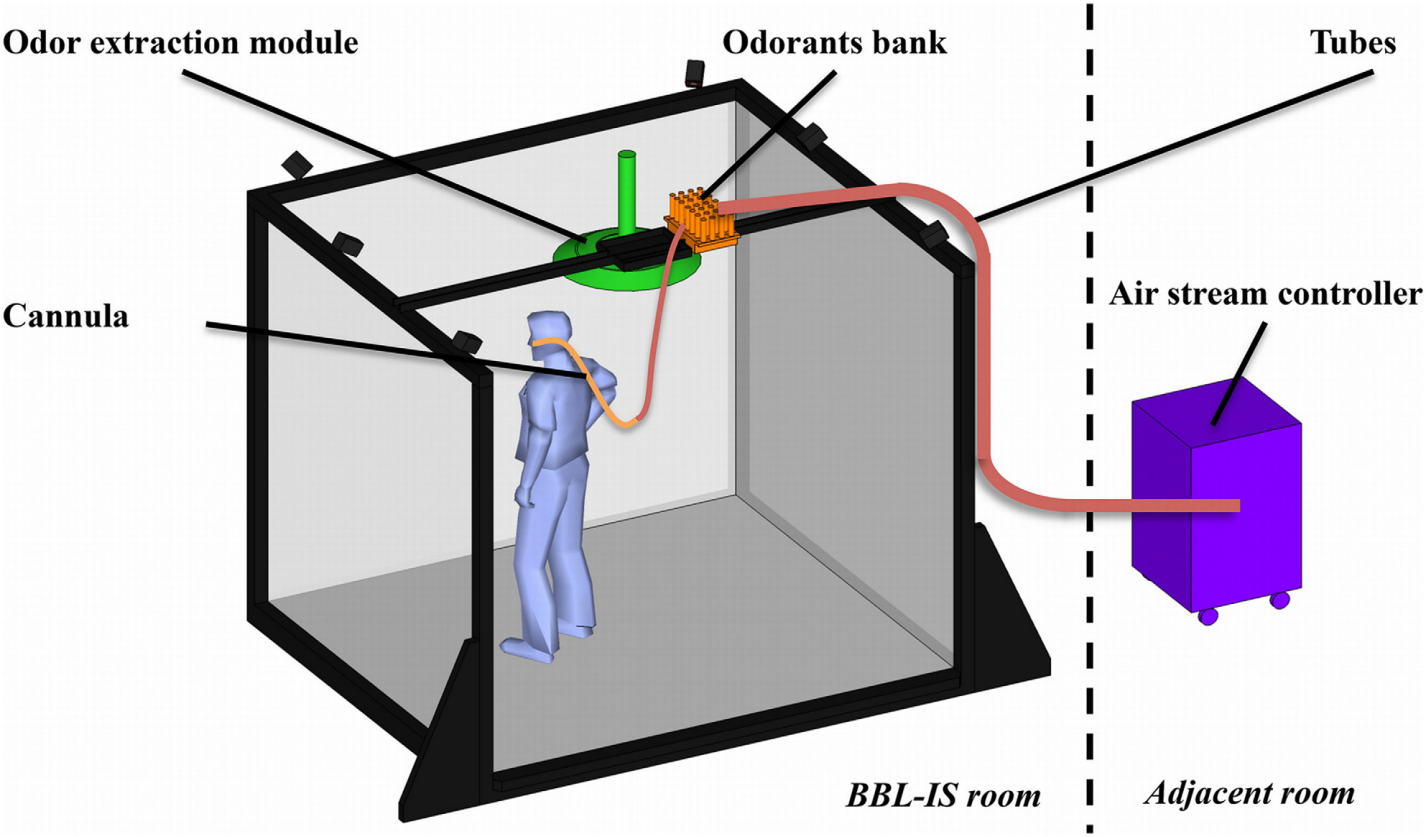
**Schematized illustration of the OD integrated in the BBL-IS**.

The OD is connected to the medical air supply of the building (an internal compressor is also available) and is filtered using charcoal filters. Thus, no extraneous odorant or particle can contaminate the airstream that enters in the main flow meter, which can be manually adjusted according to the experimental design. Then, the air is distributed to different solenoid valves. Opening and closure of the valves are rapidly controlled via a relay controller card (National Control Device®, ProXR RS-232 E3C). The control of the card is performed via a custom-developed library that can be run by various software (e.g., Eprime®, Matlab® and Unity®). In the non-active state, the inter-stimuli interval (ISI) air valves are open so that clean air is delivered to the nose. During odor delivering, ISI valves are automatically switched off and the corresponding odor valves are switched on. Consequently, manipulation and control are simple, even with up to 28 different odors. Each channel's flow rate can be manually regulated by limiters and is usually fixed around 1 l.min^−1^. This low-intensity airflow simplifies the system because it is no longer necessary to humidify or to heat the air for participants' comfort (e.g., Lorig et al., 1999). Since both ISI and odorant flows are setup to the same level, the flow rate perceived in the nose remains constant (see below for an empirical demonstration); only the noise coming from the valves might give external clues about the olfactory stimulation. In order to avoid this issue, the airstream controller is situated outside the experiment room (see Figure 2).

Airflows leaving the airstream controller are conveyed through small diameter polyurethane plastic tubes (inside diameter: 2.5 mm) of equal length to ensure accurate timing of odorant delivery. They reach the bank of odorants made up of custom-made glass vials positioned on the top of the IVR system via custom-made support (see Figure 2). Odorant vials in this design are made of small glass cylinders (22 mm of diameter × 120 mm high). Odorants are placed inside each vial using pen's tampon (Burghart® GmbH) filled with different quantities of pure odorants or odorants dissolved in solvents (e.g., propylene glycol or mineral oil). Odorant molecules evaporate in the vial, creating a headspace of constant volume. The availability of many glass vials allows the use of the same molecule at many different concentrations or many different molecules at a known concentration. This design also intends to mimic a natural environment in which different odors are present separately or as a combination.

Glass vials are positioned as close as possible to the participants' nose (Figure 2) in order to reduce the cross-contamination between odorants. The proximity between the nose and the odorants, associated with the small diameter of the tubes connected to the air stream controller allow the rapid delivery of odorant molecules into the participants' nose within a short delay (as empirically demonstrated below). All tubes (PTFE) are gathered using Y push-in fittings (Festo®) and the final delivery piece is a light and easy replaceable nasal cannula directly positioned at the entrance of the nostrils. This light device tends to be unnoticed by users after a few minutes and delivers small quantities of odorant directly into the nose, minimizing the pollution of the ambient air. Cannulas' prongs are sized so as not to obstruct the nose, allowing the subject to breathe normally.

In order to avoid olfactory contamination of the room by the odorant releases, a first air-extractor (125 m^3^.h^−1^, not represented in Figure 2) is located on the ceiling of the room and guarantees a global air renewal. A second air-extraction system is positioned close to the participants' head without hindering their movements. This module is composed of a collector shaped as a flattened cone (diameter 50 cm) linked to the global air extraction system of the building (125 m^3^.h^−1^). Room temperature is regulated around 22°C.

To summarize and as illustrated in Figure 2, the OD setup comprises four parts: the bank of odorant located in the BBL-IS as close as possible to the user; the air stream controller placed in an adjacent room to the BBL-IS; the tubes connecting the air stream controller to the bank of odorants and then to the nose of the participants; and the odor extraction module located above the BBL-IS.

We set up the olfactory device to be safe and comfortable for the subjects, to deliver air with or without odorants at a constant rate in a reliable and reproducible manner. The switch between odors and inter-stimulus air should be almost instantaneous without other perceptible stimuli. We tried to reduce cross-contamination between odorant streams and odorant contamination of the experimental room. Our OD is also set up to deliver various and easily changeable odorants in a controllable and easy way. To demonstrate that we reached those objectives, we present a couple of validation studies (see section Performance Tests) after introducing the immersive environment.

### The “BBL-is” system

**Figure 3:**
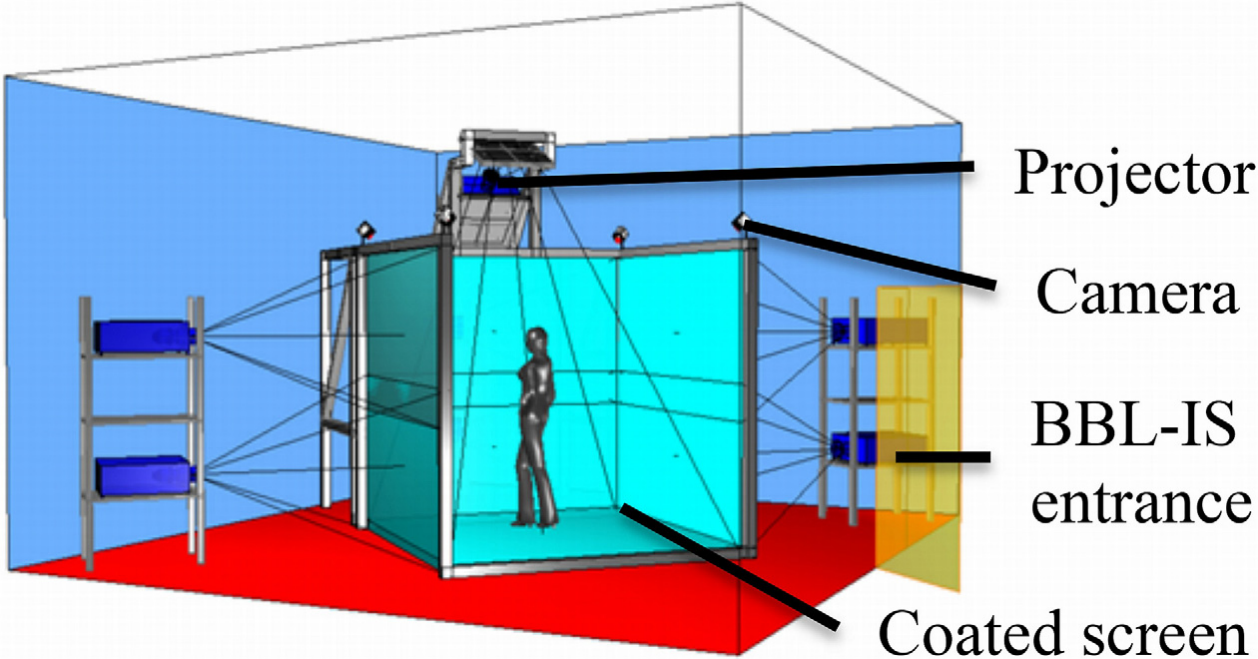
**Schematic representation of the “BBL-IS” system**.

The IVR system, which provides a 3D, immersive and fully interactive visualization environment, is installed in the Brain and Behavior Laboratory (BBL) of Geneva. This system, called BBL-IS (BBL-Immersive System http://bbl.unige.ch/ResearchModules/BBL-IS.html) is shaped as a room-sized cube, using four walls as screens on which images are projected by several synchronized video projectors (see Figure 3).

#### Technical specification

The BBL-IS has 4 sides presenting seamless and perspective coherent 3D images for user wearing IVR glasses (Figure 3). Seven video projectors (Digital Projections®, TITAN QUAD 3D WUXGA) project high-resolution images (1920^*^1200 pixels) at 120 frames per second. Projection is performed on the four acrylic coated screens (DaLite®, 2.8 m wide and 2.4 m high) with a high contrast ratio and brightness (1600 cd.m^−2^ per screen). An optical motion tracking system composed of eight infrared cameras (Vicon®, Bonita 3) is used to capture the participant movements. These movements' parameters are integrated in the virtual scenario to create a fully interactive environment. In our configuration, an infrared reflective sensor is positioned on the virtual reality glasses to mimic the position of the nose. This position will be recorded on line and used to trigger the odorant delivery. The environment rendering is provided by a cluster of workstations. Further technical information is available at http://bbl.unige.ch/ResearchModules/BBL-IS.html.

This platform allows researchers to benefit from the state-of-the-art in virtual reality for creating and using immersive scenarios. More particularly, fully controlled manipulations of visual, auditory and olfactory stimulations coupled with the possibilities to track eyes, head and body movements of one participant allow scientists to investigate complex behaviors and emotional responses in realistic scenes.

### Integration of the olfactory device

#### Control of odorant delivery

**Figure 4:**
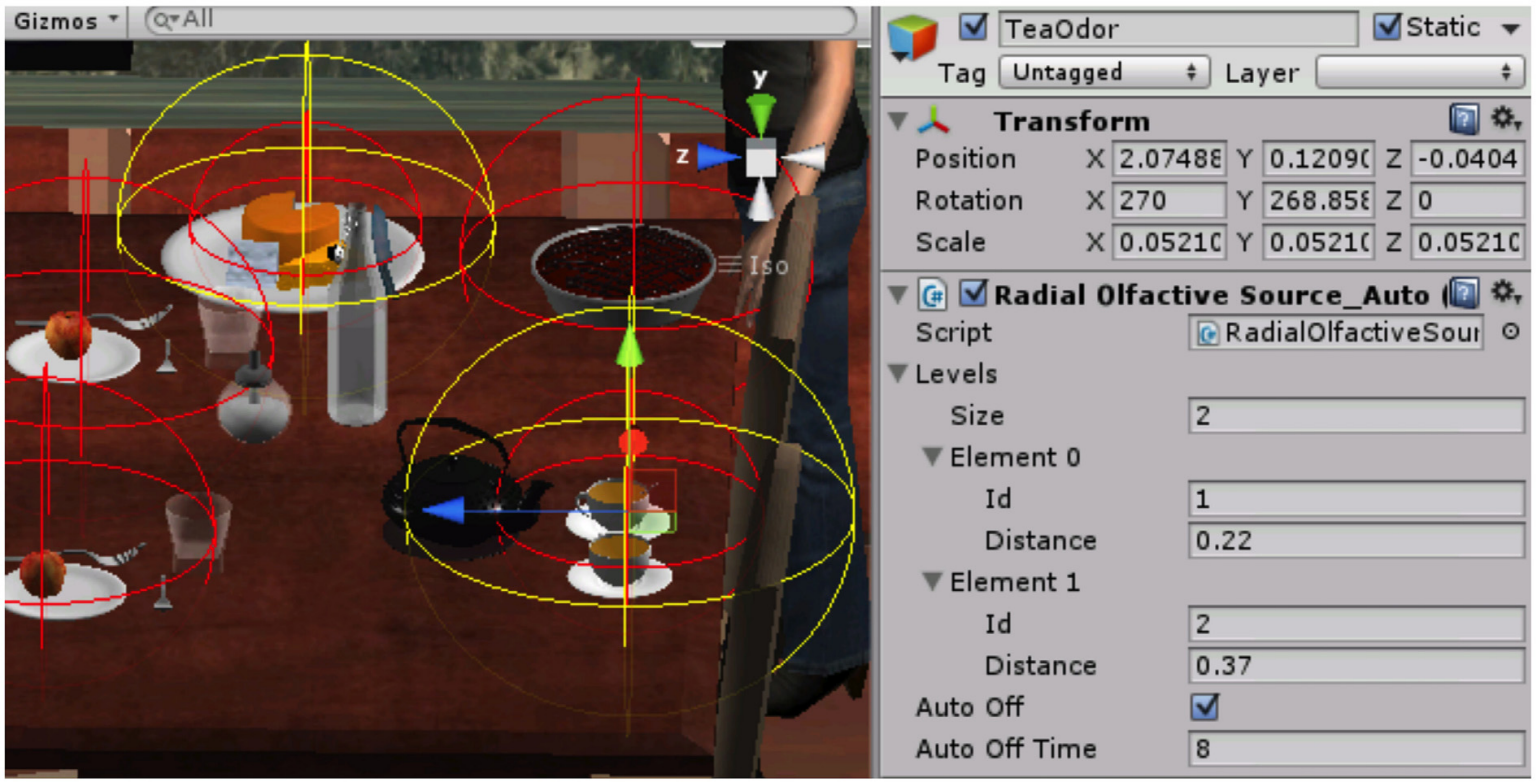
**Screenshot of the Unity3D editor while building a virtual kitchen with odorant objects (e.g., tea, cheese etc.) and 3D olfactory volumes (in red and yellow)**.

The control of the OD can be performed manually via a simple software or computer-controlled via a custom made software toolkit (Geneva Virtual Reality Elements, GeVRE). The latter brings IVR related features to existing interactive 3D software, mainly in Unity3D®^3^. GeVRE extends the capabilities of classical 3D software by adding the features needed to build and run IVR environments (e.g., displaying synchronized images with perceivable depth, allowing cluster computing, adjusting the images perspective to the user's point of view, integrating various devices such as position tracker, haptic devices, joystick, etc.). GeVRE also provides modules for scientific studies, like event coding to synchronize external recording devices, accurate binocular gaze data recording and OD control functions. More importantly for our purpose, GeVRE allows researchers to use Unity3D® to build a complex virtual environment comprising smells. The basic principle is as follows: the researcher defines olfactory sources that possess different parameters such as the odorant type, the shape and size of olfactory volumes, and their position. All those features are defined directly using the interface of Unity3D® (Figure 4). This option makes the control of the OD easy and flexible.

More precisely, the experimenter specifies the attributes of the world that permit the definition of the complex olfactory environment: (1) the object that represents the nose for the system and that is tracked on line via the motion tracking system, (2) the type of odorant (up to 28 possibilities), (3) the position and the shape of the olfactory volume (i.e., from spheres to complex geometric figures), and (4) the behavior of the olfactory source (e.g., transient or permanent delivery, moving source).

**Figure 5:**
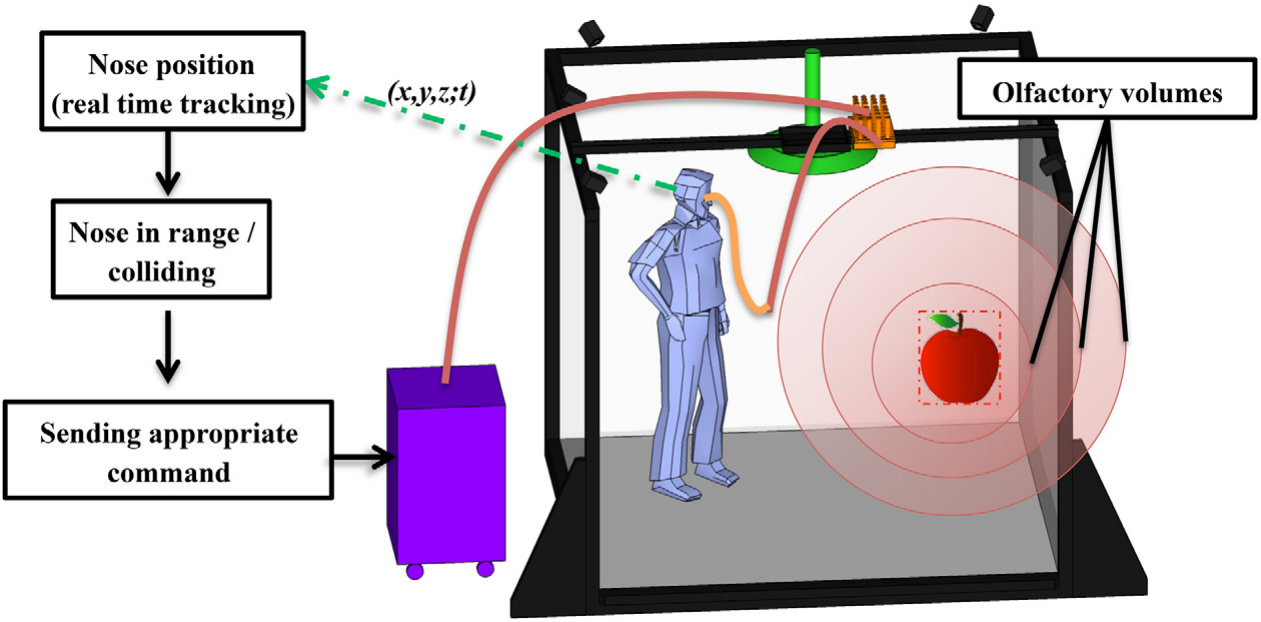
**Schema of the control protocol**.

After the communication between the OD and GeVRE has been established, an odorant is triggered according to the user's nose position estimated from the BBL-IS tracking system. When the nose enters one of the olfactory volumes defined by the user, the command is sent to the OD to deliver the corresponding odor. Multiple olfactory volumes can be used on the same object to create an odor gradient and recreate a natural scene (see Figure 5).

A custom-made dynamic link library, which provides predefined functions to send activation or deactivation commands for a specific odorant to the relay controller card, achieves the communication. One of the advantages of this GeVRE toolkit is its modular design allowing to implement another OD with ease (more information are available upon request).

## Performance tests

The objective of this report is to present an OD solution that is efficient to reliably deliver a large number of different odorants in the IVR environment in a reproducible manner, at a low and constant flow rate among all subjects, without other perceptible changes (i.e., noise or tactile) and without cumbersome apparatus attached to the participant. The following sections present results of different tests performed on the OD to demonstrate its efficiency to reliably deliver odors in the BBL-IS.

### Gas detector analyses

**Figure 6:**
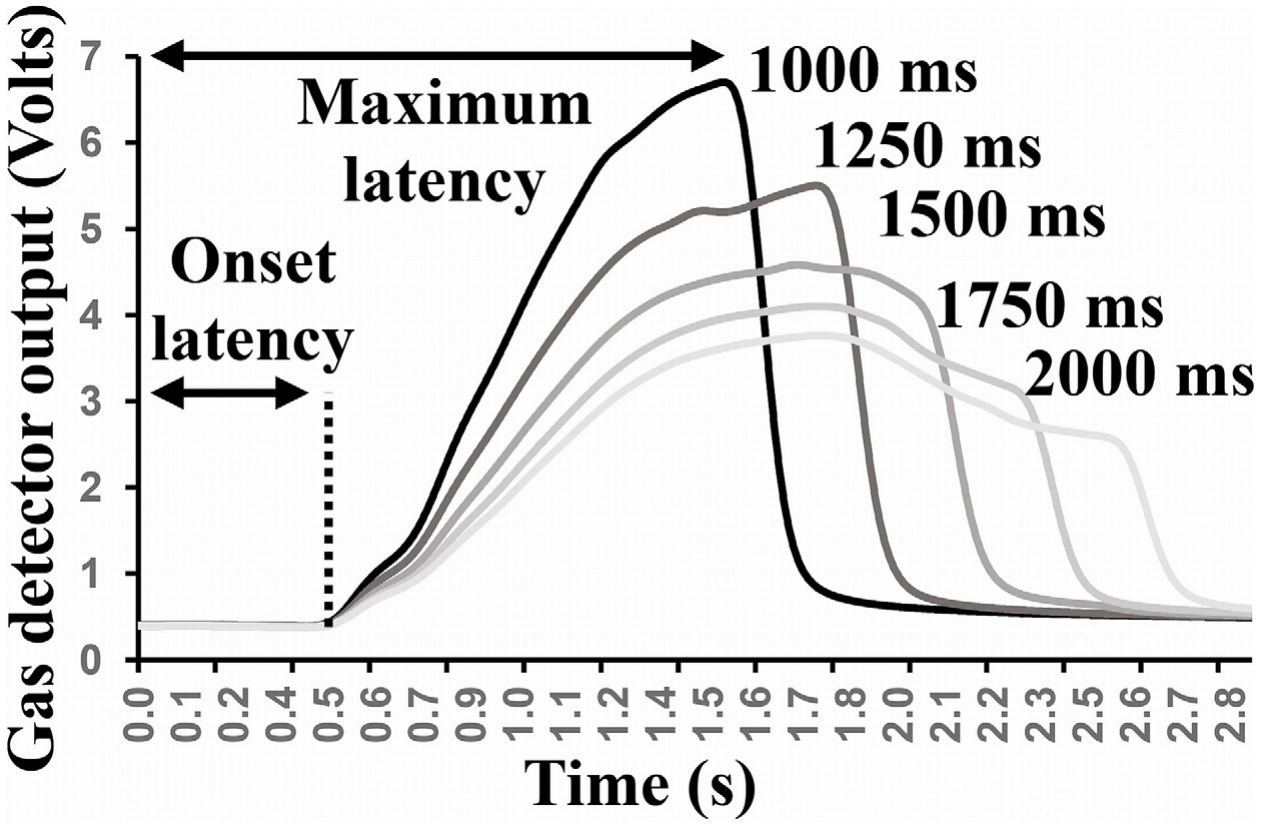
**Averaged gas detector response changes across all 50 pulses (ISI = 5 s) for each pulse duration condition**.

The extremity of the OD was connected to a photo-ionization detector (miniPID 200B, Aurora scientific inc.) which monitors concentration changes of an input gas or vapor across time, at a millisecond resolution. The output (in Volts) is proportional to the concentration of sampled compounds and was recorded for offline analyses using Biopac® system with a sampling rate of 1000 Hz. Five blocks of fifty odor pulses (classical shampoo fragrance diluted in dipropylene glycol at 10%, flow rate 1 l.min^−1^) were triggered with a constant inter stimulus interval (ISI) of 5 s. Within each block, the duration of pulses was constant and increases by 250 ms steps, beginning at 1000 ms up to 2000 ms. Those durations were chosen as they could be used inside a unique inspiration phase. Within each block and for each pulse, the latency of the response onset as well as the maximum of concentration changes and its latency were extracted with the Acknowledge® software (Biopac® System). The averaged (across the 50 trials) output responses are presented in Figure 6.

#### Latency calculation

Latency between the command to deliver the odorant and the actual delivery at the nostrils level is a key aspect of every OD. If the participants realize that the olfactory scenario being depicted is not actually occurring, they will lose the sense of presence. As participants' movements in the direction of an olfactory source can be unpredictable, the latency must be as short as possible. The concentration of the compound starts increasing at latencies varying from 433 to 455 ms after the valve was opened, reaching its maximum between 1500 and 1748 ms, depending on pulse duration. The biggest onset latency fluctuation between different pulse durations represents a time variation of around 5% of the mean. Ninety-five percent of onset latencies values are situated within an averaged time margin of ± 6.36% of the mean onset value. These analyses demonstrated that, despite the 13 m length of the tubing part and the passage of the air through the glass vials, the system is able to deliver the compounds as early as 440 ms after the triggering command. Even for long aperture durations with a short ISI (5 s) the maximum of concentration is reached during the first 2 s for this particular compound. In sum, the OD is fast enough to provide a puff within a unique inspiration phase.

#### Concentrations reliability

**Figure 7:**
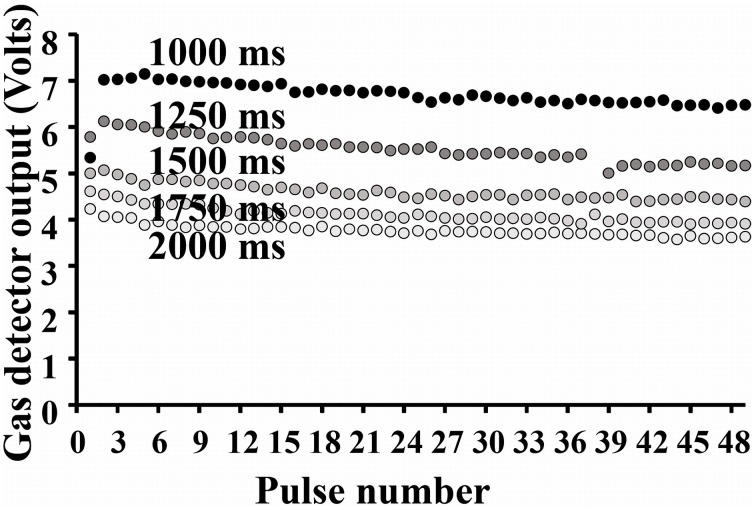
**Gas sensor output values for each of the 50 pulses as a function of the pulse duration**.

We also measured the maximum amplitude of the gas detector output signal (i.e., the maximal concentration) of the compound for each of the 50 pulses. Gas sensor output values as a function of the pulse duration are represented in Figure 7.

**Figure 8:**
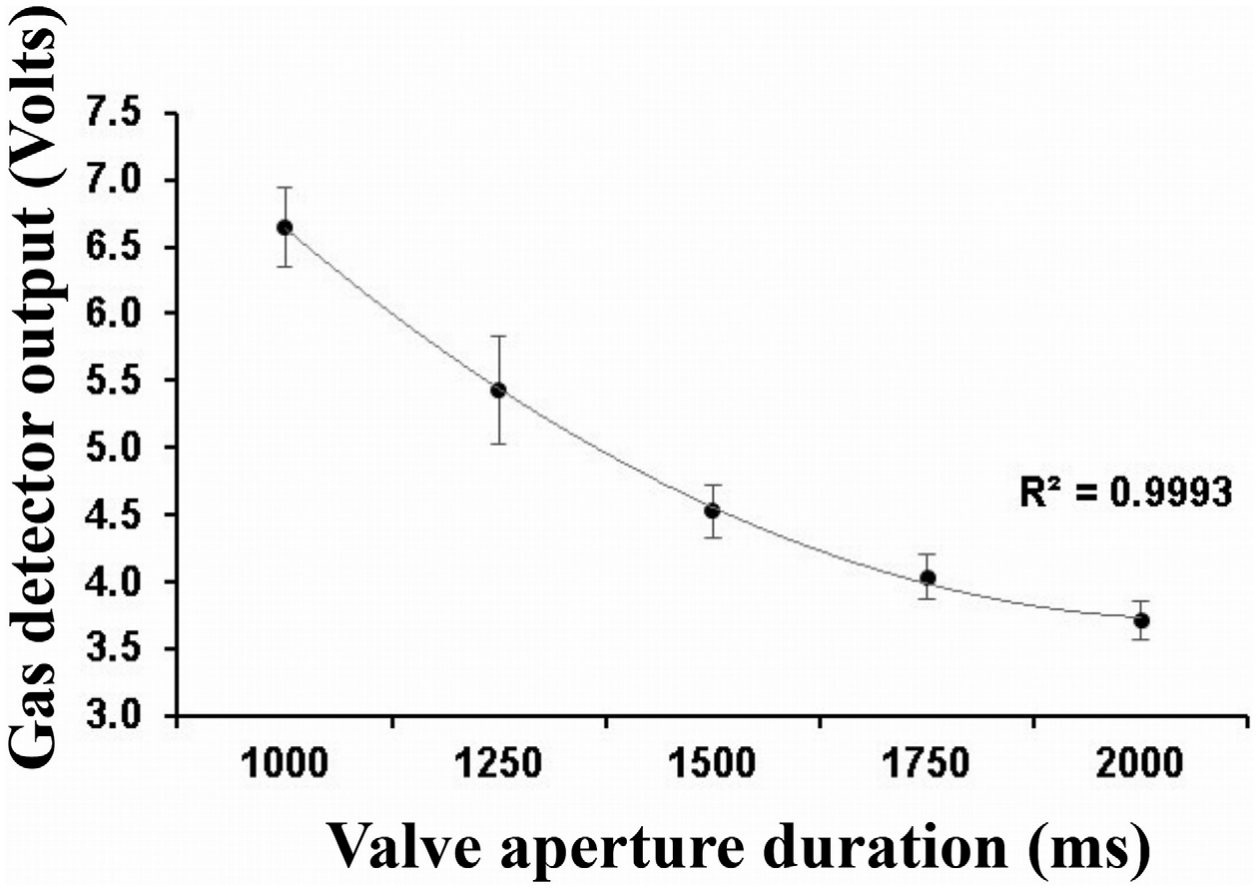
**Mean gas sensor maximum amplitude values as a function of the duration of valve aperture**. Error bars indicates the 95% confidence interval. The quadratic correlation coefficient is also indicated.

For a given pulse duration, the reproducibility of odor delivery across the successive pulses was very satisfying. Ninety-five percent of maximum amplitude values are situated within an average margin of ± 4.82% of the mean maximum value. The measures also revealed a decrease in maximum concentration available as a function of the increase in duration of valve aperture. This relation is represented in Figure 8. The high quadratic regression coefficient indicates that maximum concentration tends to stabilize as the aperture time increases. These changes in concentration as a function of aperture duration are linked to the fixed ISI we employed. Indeed, the longer the aperture duration, the longer the time needed to recover the initial headspace.

**Figure 9:**
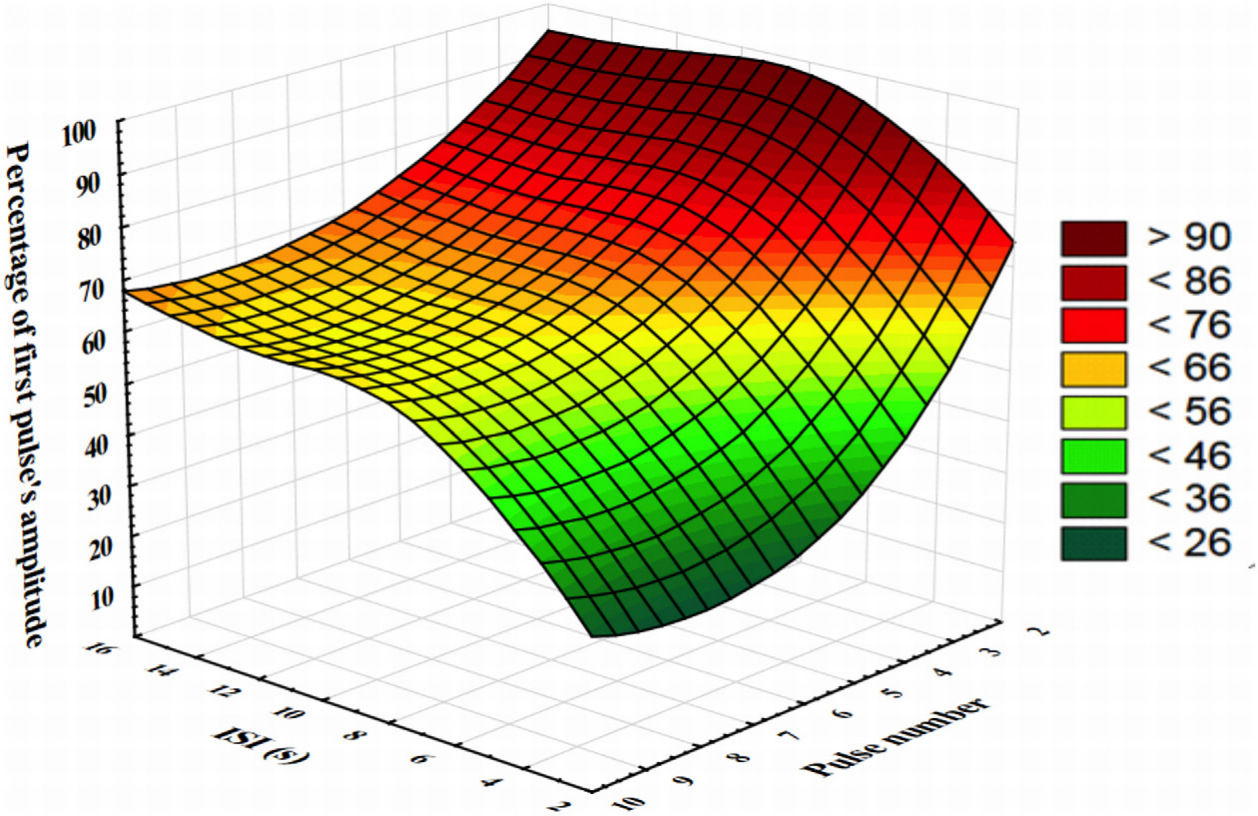
**Percentage of gas detector output changes as a function of the pulse number and the ISI**.

To address this issue, we performed a supplementary test during which we measured gas detector output values during sequences of 10 pulses of another odorant (apple aroma B diluted in dipropylene glycol at 20%, 1000 ms duration). Eight sequences with fixed ISI of 2, 4, 6, 8, 10, 12, 14, and 16 s were launched. Each new sequence was separated from the preceding one by 2 min to allow the recovery of the headspace. We then measured the maximum amplitude of the gas detector output signal during the odorant delivery. Then, for each pulse, we calculated the percentage of change according to the maximum amplitude of the first pulse of the sequence. In Figure 9, we then reported those percentages of change as a function of the pulse number and the ISI. This graph reveals that the shorter the ISI, the stronger the initial reduction in maximal signal output. For instance, 2 s ISI leads to a reduction of more than 60% in quantity of compounds after four pulses. Fortunately, those conditions are unlikely to occur in a virtual environment since it requires the participants to cross the same olfactory area every 2 s four consecutive times. Concentration reliability if far better for ISI superior to 8 s and stabilizes around 70% of the maximum quantity of compound after 5 pulses. For ISI equal or superior to 8 s and for less than 5 consecutive pulses, the quantity of compound delivered is around 90% of the quantity of the first pulse. This latter condition will constitute the majority of the situations participants will be exposed to inside the virtual environment. So as suspected, the number and the interval between consecutive pulses of odorant can have an impact on the quantity of product released. Consequently, the time the individual will spend sampling the odorant, the number of samples and the interval between consecutive samples will clearly affect the quantity of compound he will be exposed to. All those variables should be recorded to perform appropriate statistical corrections if needed.

#### Cross-contamination test

**Figure 10:**
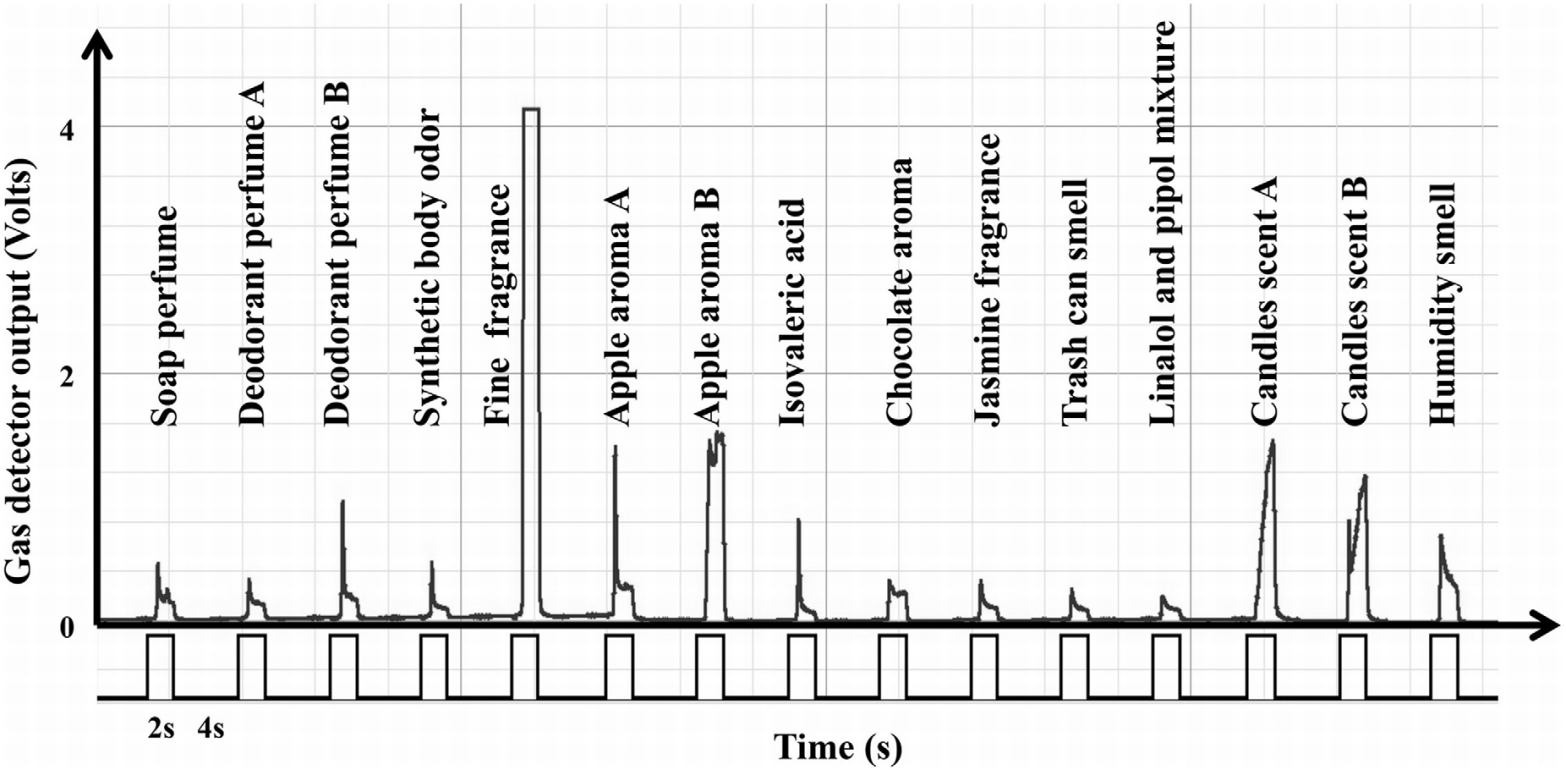
**Gas sensor output values obtained for one sequence of 15 different odorants delivery**.

**Figure 11:**
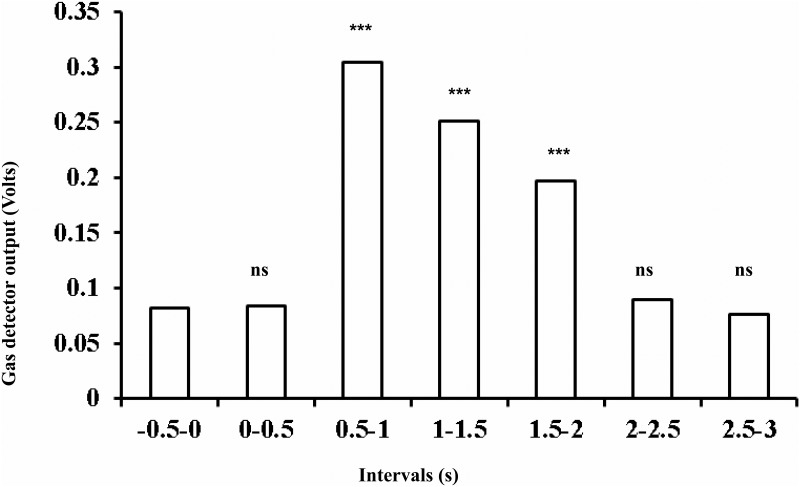
**Mean gas detector output values for the six 500 ms periods and the baseline**. ns, non-significant, ^***^ = *p* < 0.001

Another key issue in olfactory displays fabrication is to minimize cross-contamination. Cross-contamination corresponds to the contamination of one odorant by remaining traces of the preceding one. This cross-contamination depends on the compound properties (i.e., volatility, interaction with tubing material) and the ability of the system to evacuate remaining odorant molecule during the ISI. Observed gas detector values at the closure of the valves seem to reach their pre-aperture values after a delay of around 500 ms (see Figure 6). This descriptive result suggests that any other odorant delivered after this recovery period is unlikely to be contaminated by the preceding odorant. To investigate this point more thoroughly, we measured the gas detector output values for 15 different odors (see Figure 10 for the name of the odorants). Each odorant was delivered 10 times at 1 l.min^−1^ flow rate for 2 s with an ISI duration of 4 s. Gas sensor output values obtained for one sequence of 15 odorants delivery are represented in Figure 10. The gas detector output values averaged 500 ms before each valve aperture were used as baselines for each trial. For each odorants, we averaged the gas detector values within successive 500 ms periods across the 10 trials during the 3 s following valve aperture. The resulting gas detector mean values averaged across odorants are represented in Figure 11. Non-parametric tests (Sign Test) were used on those averaged values to statistically compare each of the 6 periods to the baseline. To address the problem of multiple comparisons, we applied the Bonferroni correction to all analyses (for *n* = 6 comparisons, the new significance level is set to 0.05/*n* = 0.0083). Results indicated that the mean gas detector values were significantly different from the baseline for 0.5 to 1 s, 1 to 1.5 s, and 1.5 to 2 s periods (all *Z*_s_ = 3.61; *p*_s_ < 0.001). Gas detector values obtained just before and just after the delivery of the odorant are not significantly different from baseline values (see Figure 11). This result indicates that the level of ionization obtained just after the delivery of the molecules is similar to the level before, rendering the cross-contamination highly unlikely. However, since cross contamination could depend on many other factors like the valves' aperture time, this test should be systematically performed before any new experiment.

### Psychophysics of flow detection

The objective of this test was to investigate whether participants were able to detect flow changes potentially occurring during odor delivery at 1 l.min^−1^. Indeed, when an odor is delivered, the OD switches from the inter-stimulus airflow to the odor flow. This change could produce differences in the net flow that may create a perceptible tactile stimulation in the nose. In order to control this potential problem, we performed a supplementary flow detection task.

#### Procedure

Twelve volunteers (29.8 ± 6.8 years old; 5 females, 7 males) performed the detection task. When requested, they had to concentrate on any sensation that they could perceive in their nose while they were connected to the OD receiving the different stimulations (airflow fixed at 1 l.min^−1^). After each trial, participants had to report to the experimenter whether they perceive any change in their nose sensations. They were presented with three kinds of trials: (1) no change at all, (2) opening of a valve with the same airflow as the ISI airflow for 1 s or (3) opening of one valve with the pure odorant (orange aroma) for 1 s. The task comprised a total of 10 trials per condition presented at random.

#### Analysis

For each individual, we calculated a sensitivity measure (d′) based on hit rate and false alarm rate. A hit was recorded when the opening of a valve occurred and was detected by the participant, a false alarm was recorded when no change occurred but was falsely detected by the participant as such. The greater the participants discrimination abilities, the higher the d′ values (zero meaning no discrimination).

#### Results

**Figure 12:**
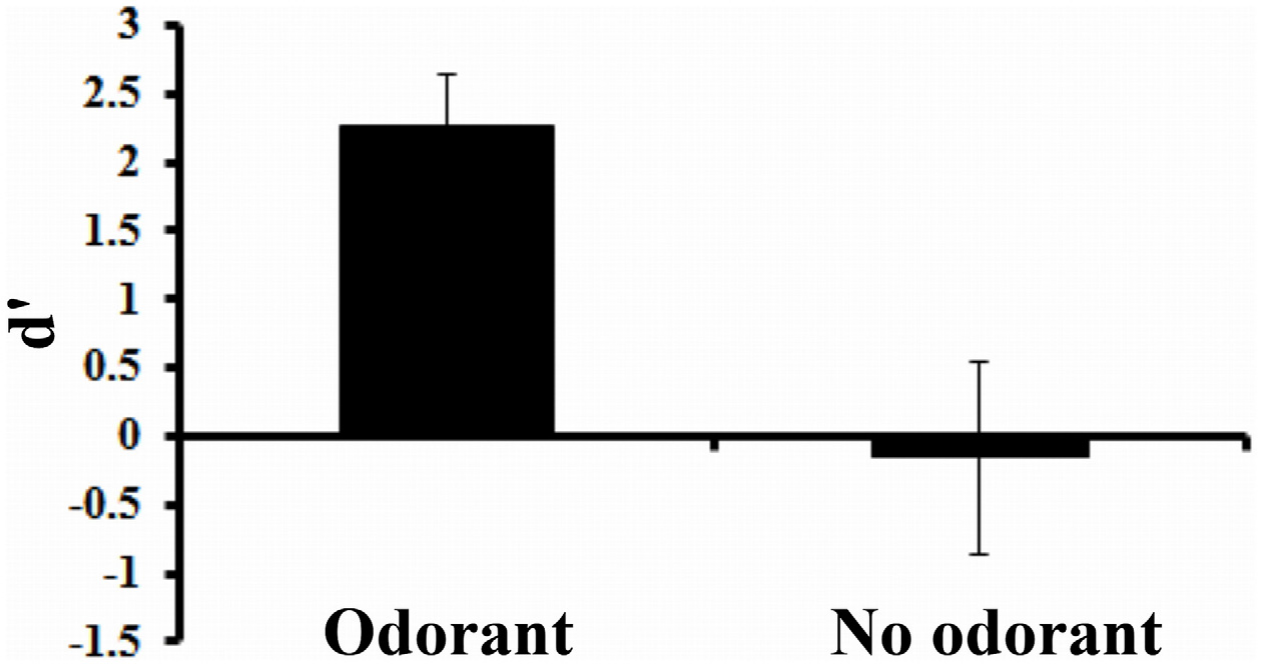
**Mean d′ (± *SD*) in the two conditions**.

The mean d′ calculated for each condition (odorant and no-odorant vial) is presented in Figure 12. In the no-odorant condition, d′ did not differ from zero (Test of means against reference constant, *t*-value = −1.45; *df* = 11; *p* = 0.18). This result indicates that participants were not able to perceive a change during valve switching. By contrast, when the odorant is added in the flow, participants clearly detect a difference (test of means against zero, *t*-value = 10.81; *df* = 11; *p* < 0.001). This psychophysical experiment shows that under the normal condition of use (ISI airflow similar to odor airflow), participants should only detect changes in the nose due to odorants.

### Spatialized detection test

At the perceptive level, cross-contamination results in the perception of a mixed odor while different odorants are actually delivered at separate moments in time. To investigate whether the present OD suffers from cross-contamination at a perceptive level, we developed an olfactory two-alternative forced-choice task (2AFC). Since the originality of our platform is to be able to spot odor sources in 3D environments, we focused on consequences of cross-contamination on the ability to discriminate odor sources in space. We aim at testing whether different virtual olfactory objects can be rapidly and accurately distinguished from one another, even if they are close in the virtual space as could be real olfactory sources. This distinction can be really impaired if there is cross-contamination in the OD.

#### Procedure

Nine healthy volunteers (31.6 ± 4.3 years old; 5 females, 4 males) participated in the test. After being connected to the OD via the cannula, they were immersed into a simple dark immersive virtual environment made of a floor composed of a white grid pattern (a video presenting the test in the BBL-IS is presented at http://www.affective-sciences.org/virolfac). Two virtual white spheres with a radius of 50 mm, and respective center points located at 150 mm distance from each other were presented in the virtual environment. Those olfactory virtual spheres were thus separated in space by only 50 mm. Participants move into the virtual world to reach the spheres and were requested to smell each sphere rapidly and to determine which one contained a randomly assigned target odorant. The two odors (orange and soap-like) were randomly assigned across the trials. Each odorant was delivered for 500 ms. Participants had to select the correct sphere with a remote control. After each trial (*n* = 8), participants had to wait 20 s before another couple of spheres appears in the virtual world.

#### Results

The binomial distribution is used to set our criteria for the correct odor detection; specifically, the minimum numbers of correct judgments to establish significance for the 2AFC test (one-tailed, α < 0.05, probability of guessing *p* = 1.2) is calculated. For *n* = 8 tests, the minimum numbers of correct judgments is seven. Six participants obtained eight correct answers and three participants seven correct answers. All participants could discriminate the target odor above chance. In total, the percentage of correct response was 95.83% (± 6.25). For indicative purposes, we also calculated the time elapsing between the samplings of the two spheres. Participants spent on average 6.48 s (± 2.28) to smell the two spheres. All the participants reported noticeable and clear perception of the two odorants.

#### Conclusion

This psychophysical test reveals that participants accurately distinguish two different olfactory objects separated in space by only 50 mm. Participants performance and subjective reports indicate that cross-contamination is very unlikely to occur with this olfactory design. However, as the cross-contamination is also dependent on the molecules used, this should be formally measured before each experiment.

## Discussion

The objective of this report was to present an olfactory display connected to an IVR system that is efficient to deliver a large number of different odorants in the virtual environment: (i) at a low and constant flow rate among subjects and without other perceptible changes (i.e., noise or tactile sensations), (ii) with limited cross-contamination between odorant streams, and (iii) with an easily and controllable interface. The platform, combining a new state-of-the-art BBL-IS system and a high-performance OD, offers excellent characteristics for researchers in behavioral sciences. As demonstrated by the different tests we performed, the OD rapidly (~ 440 ms) releases various kinds of compounds (up to 28) over multiple trials, with almost no contamination from one trial to the other, at known timings, localization and strength, and without noticeable supplementary noise or tactile stimulations in the nose. By being shorter than the inspiration and the expiration phases, those latencies would allow to synchronize the odorant delivery and the participant breathing pattern.

Several caveats and limitations of this olfactory platform need to be mentioned. The first important limitation of the system is its cost. The availability of such IVR environment is far from being worldwide. Only few research centers are equipped, increasing the difficulty to reproduce the results and potentially decreasing the scope of the conclusions. One can only hope that the current and future technological advances in this domain will allow easier and less expensive immersive virtual environment implementation in many different laboratories. A second important point is that it remains unclear whereas such complex experimental settings will really help researchers to answer fundamental and/or applied questions, when compared to classical experimental setups. Although a benefit is clearly expected, further quantitative and qualitative studies will be needed to directly compare those two situations. A third limit worth mentioning is that the reliability of the OD we present in this report is highly dependent on factors that are modified as a function of the research question and the experimental procedure. For instance, increasing odorants' concentration and the number of olfactory sources in the virtual environment will increase the likelihood of cross-contamination. This should be controlled with gas detector analyses as well as psychophysical tests before each new experiment. Moreover, we demonstrated that concentration release is dependent on the duration of the stimulation, the number of successive presentations of the same odorant and the interval between those presentations. Since in most virtual reality applications, participants' sampling behavior will condition those parameters, it will thus be necessary to measure them during the experiment.

Being able to provide visual, auditory and olfactory stimulations in a fully controllable and close to reality environment should allow researchers to study complex and multisensory interactions. For instance, a key research question in olfactory literature remains how and to what extent chemosensory preferences can be modulated. It is now well accepted that needs, goals, values, learning and exposure deeply influence odorant perception and preferences (see Coppin and Sander, 2011 for a recent review). Future studies could be conducted in the immersive virtual environment to investigate more thoroughly the role of the perceptual changes as well as social interactions on odors evaluation or emotional reaction. The richness and the closeness to reality quality of the environment should help researchers to better understand how the different senses work together to elicit subtle, personal and variable emotional reactions or to shape implicit or explicit olfactory memories. All those research questions can be addressed simultaneously at the cognitive, behavioral and physiological level. For instance, the platform can easily integrate and be synchronized with wireless psychophysiological recording systems. In addition to the recording of the breathing pattern that is so important in olfaction research, several peripheral psychophysiological measures (e.g., electromyography, electrocardiography, electrodermal activity, skin temperature) can be recorded and analyzed off line or even used in real-time to modify virtual world. Associated with covert behavioral responses like action tendencies, investigation times, eye position and also participant's overt subjective responses, this platform constitutes a unique opportunity to study complex, multi-level phenomena like emotions.

In addition to the fundamental research questions that could potentially be addressed with such platforms, immersive reality olfactory environments offer a potentially very power tool for clinical applications. For instance, promising virtual reality therapy exists to reduce pain and anxiety of burn victims (Morris et al., 2009), to help restoring memory deficits in people with acquired brain injury (Yip and Man, 2013) or to enhance behavioral treatments of compulsive eating related disorders (Cesa et al., 2013). Using the powerful effect of odors on moods and emotions (Schiffman et al., 1995; Rétiveau et al., 2004) during those virtual therapies could increase their efficiency. Other authors have already stressed how useful could be the inclusion of olfaction in immersive virtual environment for virtual therapy in post-traumatic stress disorder resulting from military assault or combat (Pair et al., 2006).

Virtual reality environments coupled with olfactory displays could foster new researches in development departments of many companies worldwide and revolutionize many steps of a product design. For instance, sensory research departments of fragrances and flavors aim at providing products that people prefer, and at understanding how emotions are elicited and measured. Given that direct product experience is generally the optimal method for consumers to learn about products, looking for verisimilitude in sensory research is a key objective. Mimicking normal everyday conditions in a controlled virtual environment could increase understanding how the senses work together to create the overall product experience including emotional experience that fills a typical person's life.

The replication of everyday life environments in laboratory experiments is crucial in behavioral sciences because it directly improves the ecological validity of the results, especially when complex interactions are concerned. Virtual reality environments provide both the complexity of the real world that could elicit vivid human experiences and the control of the experimental variables that is a prerequisite to produce reliable conclusions in behavioral research. In that sense, every attempt to include the olfactory modality in virtual environments should be actively fostered.

### Conflict of interest statement

The authors declare that the research was conducted in the absence of any commercial or financial relationships that could be construed as a potential conflict of interest.

## Footnotes

^1^The sense of presence is defined by the fact that participants forget that their perception is mediated by technology. Main criteria for a good sense of presence are; a) the sensation of being in a real place (place illusion) and b) the illusion that the scenario being depicted is actually occurring (plausibility illusion; Slater, 2009).

^2^In describing our apparatus, we will use the term “OD”, defined as “… *a collection of hardware, software, and chemicals that can be used to present olfactory information to the virtual environment participant*” (Barfield and Danas, 1995).

^3^www.Unity3D.com.

## Supplementary material

http://www.controlanything.com/
